# Estrogen receptor subtype mediated anti-inflammation and vasorelaxation *via* genomic and nongenomic actions in septic mice

**DOI:** 10.3389/fendo.2023.1152634

**Published:** 2023-05-17

**Authors:** Luyun Zhang, Hanxing Wan, Mengting Zhang, Wei Lu, Feng Xu, Hui Dong

**Affiliations:** ^1^ Department of Pediatric Intensive Care Unit, Children’s Hospital of Chongqing Medical University, National Clinical Research Center for Child Health and Disorders, Ministry of Education Key Laboratory of Child Development and Disorders, Chongqing Key Laboratory of Pediatrics, Chongqing, China; ^2^ Department of Pharmacology, School of Pharmacy, Qingdao University Medical College, Qingdao, China

**Keywords:** ERα, ERβ, GPER, endothelium-derived hyperpolarization, inflammatory cytokines, sepsis

## Abstract

**Aim:**

Sepsis is a life-threatening disease with high mortality worldwide. Septic females have lower severity and mortality than the males, suggesting estrogen exerts a protective action, but nothing is known about the role of vascular endothelial estrogen receptor subtypes in this process. In the present study, we aimed to study the estrogen receptors on mesenteric arterioles in normal and sepsis mice and to elucidate the underlying mechanisms.

**Methods:**

Sepsis was induced in mice by intraperitoneal injection of LPS. The changes in the expression and release of the serum and cell supernatant proinflammatory cytokines, including TNF-α, IL-1β and IL-6, were measured by qPCR and ELISA, and the functions of multiple organs were analyzed. The functional activities of mouse mesenteric arterioles were determined by a Mulvany-style wire myograph. The expression of phospholipase C (PLC) and inositol 1,4,5-trisphosphate receptor (IP_3_R) in endothelial cells were examined by Western blot and their functions were characterized by cell Ca^2+^ imaging.

**Results:**

Septic female mice had higher survival rate than the male mice, and pretreatment with E_2_ for 5 days significantly improved the survival rate and inhibited proinflammatory cytokines in septic male mice. E_2_ ameliorated pulmonary, intestinal, hepatic and renal multiple organ injuries in septic male mice; and ER subtypes inhibited proinflammatory cytokines in endothelial cells *via* PLC/IP_3_R/Ca^2+^ pathway. E_2_/ER subtypes immediately induced endothelial-derived hyperpolarization (EDH)-mediated vasorelaxation *via* PLC/IP_3_R/Ca^2+^ pathway, which was more impaired in septic male mice. E_2_/ER subtypes could rescue the impaired acetylcholine (ACh)-induced EDH-mediated vasorelaxation in septic male mice.

**Conclusions:**

E_2_ through ER subtypes mediates anti-inflammation and vasorelaxation *via* genomic and nongenomic actions in sepsis. Mechanistically, activation of endothelial ER subtypes reduces proinflammatory cytokines and induces EDH-mediated vasorelaxation *via* PLC/IP_3_R/Ca^2+^ pathway, leading to amelioration of sepsis-induced organ injury and survival rate.

## Introduction

Sepsis is a life-threatening disease caused by a comprehensive systemic inflammatory response to infection. Despite substantial advances in the management of sepsis, the mortality of severe sepsis remains high. As sepsis progresses, severe pathological damage occurs to organs, including the kidney, liver, lung, heart, and intestine, et al. Multiple organ dysfunction is the most common cause of morbidity and mortality in sepsis. (1) Some epidemiological studies revealed the gender differences in sepsis, in that females have lower severity and mortality in sepsis. (2, 3) In addition, several clinical epidemiological studies reported that females are less susceptible to posttraumatic infections and multiple organ failures. (4) A large body of animal studies also corroborated the opinion. (5) Recently, there has been renewed interest in gender dimorphism in the morbidity and mortality of sepsis. In fact, it was found that septic females have lower incidences of morbidity and mortality compared to septic males. (6, 7). A challenging problem that arises in this domain is what contribute to this sex differences in sepsis. Based on this phenomenon, some researchers attribute the gender differences in sepsis to different hormone levels between males and females. (5, 8) Compared to males, females have a high concentration of estrogen and progesterone. As the most predominant and potent endogenous estrogen, it is vital to investigate the mechanism of 17β-estradiol (E_2_) in sepsis. It was found that estrogen can exert protective effects on immune cells by antioxidant and anti-inflammatory in sepsis, (9) but little is known about the contribution of estrogens on the vascular functions in sepsis.

It is acknowledged that tissue microcirculation plays an unreplaced role in maintaining physiology function in healthy subjects by controlling blood flow perfusion to human tissues/organs. Microcirculation dysfunction can result in tissue/organ hypoperfusion, leading to multiple organ failures. Sepsis is an acute inflammatory disease accompanied by vascular endothelial cell dysfunction. (10) An endothelial dysfunction damages the relaxation functions of the blood vessels, leading to microcirculation dysfunction to promote the deterioration of sepsis. (11) Therefore, it is of great clinical significance to improve tissue microcirculation in sepsis.

The resistance arteries play a determinant role in vascular resistance and blood flow. The relaxation function of resistance arteries is regulated by multiple mechanisms, including vascular endothelium cells (VEC), vascular smooth muscle cells (VSMCs), and the autonomic nervous system. Among them, vascular endothelium cell plays a vital role in modulating vasorelaxation. It is well-established that vascular endothelial cells can generate three relaxing factors, including nitric oxide (NO), prostacyclin (PGI_2_) and endothelium-dependent hyperpolarization (EDH). (12) It was found that NO and PGI_2_ predominantly regulate vasorelaxation of large blood vessels. In contrast, EDH predominantly regulates vasorelaxation of resistant arterial. (12, 13) In addition, when NO-induced vasorelaxation is impaired, EDH can compensate for the impaired relaxation function to some extent, further indicating the importance of EDH in regulating resistant vessels. (14) However, the contribution of EDH to E_2_-induced vasorelaxation of resistant arteries is largely unclear. It was found that E_2_ can modulate the expression of the inflammatory factors in immune cells to exert beneficial effects. (9, 15) Although inflammatory factors can impair vascular functions to prompt the development of pathophysiological progression,(16) it remains a mystery for E_2_ to modulate inflammatory factors in vascular endothelial cells.

There are three estrogen receptors (ER): estrogen receptor alpha (ERα), estrogen receptor beta (ERβ), and guanine nucleotide-binding protein-coupled estrogen receptor (GPER/GPR30). E_2_ can bind with estrogen receptors to exert various physiological effects through genomic and nongenomic pathways. (17) The genomic pathways, termed as a classical mechanism, involve E_2_ interacting with ERα or ERβ of the nucleus/transcription factors to modulate the expressions of specific genes. (18) In regard to nongenomic pathways, estrogen can activate the estrogen receptors localized to the plasma membrane or endoplasmic reticulum more rapidly to exert immediate responses, such as influences on Ca^2+^ levels or kinase activity. (19, 20) These receptors are widely distributed in cardiomyocytes and blood vessels. (21) Also, E_2_ plays a positive role in the cardiovascular system to affect vasomotion and tissue perfusion. (22) It was found that activation of ER can modulate physiological functions *via* multiple pathways, including PI_3_K/AKT/mTOR, RAS/RAF/MEK/MAPK and PLC/IP_3_R pathways, et al. (17) However, few studies have determined whether ER protects against microvascular dysfunction in sepsis; and if so, what the underlying mechanisms are.

Since mesenteric arterioles are a generally accepted model of resistance vessels, (23) they play a vital role in regulating tissue perfusion and blood pressure. In the present study, we applied mesenteric arterioles and VEC to investigate the roles of three ER subtypes in vascular health and sepsis. Interestingly, we found that ER can modulate EDH-mediated vasorelaxation of normal mesenteric arterioles via PLC/IP_3_R/Ca^2+^ pathway; however, ER in VEC exerts anti-septic actions via genomic and non-genomic actions in sepsis.

## Methods and materials

### Animal study

All animal procedures were conducted according to the Animal Care Committee of Chongqing Medical University. Male and female C57BL/6 mice weighing 18–22 g and 6–8 weeks old were kept in cages with a 12 h light/dark cycle. In a temperature-controlled room, the mice were provided with unlimited water and normal chow.

After 1 week of acclimation, male mice were randomly divided into the following groups: 1) Ctrl group (n = 25); 2) LPS group (intraperitoneally injected 10 mg/kg lipopolysaccharide, n = 25); 3) LPS + E_2_ group (pretreated with 0.5 mg/kg/d 17β-Estradiol subcutaneously for 5 days + at the sixth day only intraperitoneally injected 10 mg/kg lipopolysaccharide, n = 25); 4) E_2_ group (treated with 0.5 mg/kg/d 17β-Estradiol subcutaneously for 5 days + at the sixth day intraperitoneally injected the same volume saline instead of lipopolysaccharide, n=25). Female mice were randomly divided into the following groups: 1) Ctrl group (n = 25); 2) LPS group (intraperitoneally injected 10 mg/kg lipopolysaccharide, n = 25).

At the seventh day (at 24h after LPS intraperitoneally injection), lung tissues were collected for H&E staining, the mesenteric arteries were separated for vascular function, and the blood samples were collected from the orbital sinus. Serum was collected by centrifugation at 3,000 rpm for 10 min at ambient temperature after blood coagulation for 30 min. Lungs in the experimental groups were collected after 24 h after injection of LPS for further investigation.

### Animal survival study

Animal survival was determined over 120 hours. The observation was performed every 12 h on the first day and every 24 h on the following days. All remaining animals were humanely killed at the end of the experiment.

### H&E staining

The fresh lung tissues were fixed in 4% paraformaldehyde (PFA). Then, the samples were gradually dehydrated and embedded in paraffin. After that, the samples were cut into 3 μm sections and fixed on a glass slide. The sections were stained with hematoxylin and eosin. Histological analyses were evaluated using a Leica DM 2700 P microscope (× 100; × 200; Leica Microsystems GmbH, Wetzlar, Germany).

### Functional assessment

The serum levels of aspartate transaminase (AST) and alanine transaminase (ALT) were determined by commercial kits to assess the acute hepatic injury. The serum level of Creatinine (Cr) and blood urea nitrogen (BUN) were determined by commercial kits to assess the acute kidney injury. Serum lactate (LD) and diamine oxidase (DAO) were measured by commercial kits to assess the acute intestines injury.

### Arteriole dissection and isometric tension recordings

The mice were sacrificed by cervical dislocation. Then the mesenteric loop was dissected by a laparotomy and immediately placed in the iced Krebs–Henseleit solution. The second-order branch of mesenteric arterioles was separated under a surgical microscope. Krebs–Henseleit solution contained (mM): 118 NaCl, 11.1 D-glucose, 4.7 KCl, 1.6 CaCl_2_, 1.2 KH_2_PO_4_, 1.18 MgSO_4_, 25 NaHCO_3_ and 11.1 D-glucose.

The relaxation function of the second-order branch of mesenteric arterioles was detected by Mulvany-style wire myograph (Model 520A, DMT, Aarhus, Denmark) and Powerlab analytical system (AD Instruments, Colorado Springs, CO, USA). In the chamber bath with 5 ml Krebs solution, the mesenteric arterioles were fixed to wire myograph by two tungsten wires. In the chamber bath, the Krebs solution was continuously oxygenated with a gas mixture of 5% CO_2_ plus 95% O_2_ at 37°C with a pH of ~7.4. The fixed arterioles were kept at the tension of zero for 20 min and then normalized.

### Concentration-response curve

Cumulative concentration-response curve (CRC) to PPT (10-70 nmol/l), DPN (10-40 nmol/l), G-1 (10 nmol/l -10 μmol/l) and acetylcholine (ACh, 0.01-1000 μM) were performed in NE (5 μM)- or KCl (80 mmol/l)- preconstricted arterioles. Further, to examine the relaxation mechanisms of estrogen receptors, the relaxation function of arterioles was determined by the isometric tension tests after the fixed arterioles were treated with different activators and inhibitors for 20-30 min. Through the isometric tension tests, the relaxation effect of arterioles was unaffected by the respective vehicles (< 0.4% DMSO or H_2_O), and the contraction function of arterioles was unaffected by applied activators and inhibitors.

After normalization, the integrity of the arterial endothelium was verified by pre-constricting rings submaximally with 5 μM norepinephrine (NE) followed by relaxation with carbachol (CCh, 100 µM). We considered at least 90% relaxation response to CCh as an endothelium-intact vessel. Human hair was used to rub the intima to remove the endothelium for endothelium-denuded arterioles experiments. Then we verified the successful endothelial denudation by lack of relaxation response to 100 µM CCh (≤10%) and continued the next experiments. Only one experimental curve was proved for each preparation.

### [Ca^2+^]_cyt_ measurements in HUVEC

In physiological salt solution (PSS), a single HUVEC grown on coverslips was loaded with 5 μM Fluo-4,AM at 37 °C for 60 minutes and then washed for 20 minutes. Afterward, the coverslips were placed in a perfusion chamber on a Nikon microscope stage. Fluo-4 AM fluorescence ratio excited at 488 nm over time and detected by an intensified charge-coupled device camera (ICCD200) and a MetaFluor imaging system. The sampling interval of Fluo-4 AM fluorescence measurements was in the range of 3 s. The PSS included (mM): 140 Na^+^, 5 K^+^, 2 Ca^2+^, 147 Cl^−^, 10 Hepes, and 10 glucose. The osmolality for all solutions was ∼300 mosmol/kg of H_2_O.

### Cell culture and treatment

Human umbilical vein endothelial cells (HUVEC; USA) were incubated in endothelial cell medium (ECM, Sciencell, USA, Cat. #1001) with 10% fetal bovine serum (Gibco, USA) plus 1% penicillin-streptomycin (Beyotime Biotechnology, China). HUVEC were routinely cultured at 37 °C in a 5% CO_2_ incubator. Cells passages 10-20 were used for experiments when cell density reached approximately 90%. In subsequent experiments, HUVEC were divided into three groups: (a) Control group: HUVEC were cultured in endothelial cell medium; (b) LPS group: HUVEC were treated with LPS (1 μg/ml); (c) LPS+E_2_ group: HUVEC were treated with LPS (1 μg/ml) plus E_2_ (10 nmol/l). To examine the mechanisms involved in the protective effects of E_2_ in sepsis, HUVEC were incubated with the following compounds: 5 nmol/l PPT, 5 nmol/l DPN, 1 µmol/l G-1.

### ELISA

Serum levels of estrogen were measured by ELISA using mouse estrogen kits (EM1501, FineTest, Hubei, China). Serum levels of the cytokines, including tumor necrosis factor-alpha (TNF-α), interleukin (IL)-1β, and IL-6, were measured by ELISA using mouse-specific kits (R&D Systems, MTA00B, MLB00C, M6000B).

HUVEC were treated with different compounds at a density of 5 × 10^5^cells/well for 24h (seeded in 12-well plates). Then cell culture supernatants were collected and centrifuged at 1000 × g at 4°C for 20 min, and supernatants were then used to determine the levels of IL-6, IL-1β and TNF-a, in accordance with the manufacturer’s instructions (Elabscience, Wuhan, China, E-EL-H6156, E-EL-H0149c, E-EL-H5548c). All samples were assayed in triplicate.

### Real-time PCR

HUVEC were treated with different compounds for 6h. Then total RNA was extracted from HUVEC using RNAeasy kit (beyotime, Shanghai, China) and cDNA was synthesized using the RT Master Mix for qPCR kit (MCE, New Jersey, USA). PCR amplification with a reaction volume of 10 μL, including 0.4 μL forward primer (10 μM), 0.4 μL reverse primer (10 μM), 5 μL SYBR, 3.2 μL RNase-free water and 1 μL cDNA was monitored by BIORAD CFX Connect Real-Time PCR Software, version 1.4.1 (Bio-Rad Laboratories, Hercules, CA, USA). The following primers were used:

GAPDH-F: GGAGCGAGATCCCTCCAAAAT
GAPDH-R: GGCTGTTGTCATACTTCTCATGG
IL-1β-F: ATGATGGCTTATTACAGTGGCAA
IL-1β-R: GTCGGAGATTCGTAGCTGGA
IL-6-F: ACTCACCTCTTCAGAACGAATTG
IL-6-R: CCATCTTTGGAAGGTTCAGGTTG
TNF-α-F: CCTCTCTCTAATCAGCCCTCTG
TNF-α-R: GAGGACCTGGGAGTAGATGAG
PLC-F: CCAAATGCGCTGGACACATTC
PLC-R: CGGACACCTCTCGGAACTCT
IP_3_R-F: CCAAGCAGACTAAGCAGGACA
IP_3_R-R: ACACTGCCATACTTCACGACA

### Western blotting

After treatment for 24h, HUVEC were washed with ice-cold phosphate buffered saline (PBS). Briefly, total proteins of cultured cells were extracted with lysis buffer (RIPA, Millipore) with 1 mmol/L phenylmethylsulfonyl fluoride (PMSF), 1 mmol/L phosphatase inhibitor cocktail and 1 mmol/L protease inhibitor. The protein concentration was determined by a bicinchoninic acid (BCA) protein analysis kit (Solarbio, Beijing, China). The total lysate was separated using 10% sodium dodecyl sulfate-polyacrylamide gel electrophoresis, and the separated proteins were transferred to polyvinylidene difluoride (PVDF) membranes. Membranes were blocked with QuickBlock™ Blocking Buffer for Western Blot (beyotime, Shanghai, China) for 20 min and then incubated with primary antibodies against the human phospholipase C Beta 1 Polyclonal antibody (PLC, 1:1000, Cat No. 26551-1-AP) and human ITPR1-specific Polyclonal antibody (IP_3_R,1:1000, Cat No. 19962-1-AP) for 12h. GAPDH was used as an internal standard. Following incubation, PVDF membranes were washed with TBST three times for 10 min. Then PVDF membranes were incubated with peroxidase-conjugated specific secondary antibody for 1 h at room temperature. Bands were visualized by chemiluminescence, representative images were acquired, and Image J software (NIH Image, Bethesda, MD, USA) was used to quantify the density of each band.

### Statistics

All experiments and data collection were proved blindly. We applied technical repetition to strengthen the credibility of the data. All results are represented as means ± SEM, with n representing the number of animals in each group of experiments. In cell Ca^2+^ imaging experiments, n represents the total cells. For animal experiments, the n is at least n=5 for each group. We performed at least three independent repeated experiments for each treatment. The cumulative concentration-response curve (CRC), maximal relaxation (R_max_), area under curve (AUC), and the concentration for 50% maximal effect (EC_50_) were determined with GraphPad Software 7.0 (San Diego, CA).

The statistical significance of differences in the means of experimental groups was determined using Student’s unpaired, two-tailed t-test, 2-way ANOVA or one-way ANOVA followed by Dunnett’s post-test or *Post hoc* tests were run only if F achieved p<0.05 (GraphPad Prism 7.0, GraphPad Software, Inc.RRID : SCR_002798), and there was no significant variance inhomogeneity. A probability value of P<0.05 was considered statistically significant.

### Reagents and antibodies

All salts were from Sangon Biotech in Shanghai. Acetylcholine, N^ω^-nitro-L-arginine and indomethacin were offered by Sigma-Aldrich (St Louis, USA). NE was purchased from GRANDPHARMA (China) co. LTD. 17β-Estradiol, LPS, Propyl pyrazole triol (PPT), MPP hydrochloride (MPP), G-1, G-15, Diarylpropionitrile (DPN), PHTPP, ouabain, TRAM-34, apamin, U73122, U73343, 2-APB and SKA-31 were supplied by MedChemExpress (MCE; New Jersey, USA), SN-6 was provided by Tocris (Bristol, UK). LiCl was supplied by Macklin (China). The human phospholipase C Beta 1 Polyclonal antibody (Cat No. 26551-1-AP) and human ITPR1-specific Polyclonal antibody (Cat No. 19962-1-AP) were from proteintech. The commercial kits of serum ALT, AST, LD and BUN were from Neobioscience (Jiancheng Bioengineering Institute, Nanjing, China). The commercial kit of serum DAO was from Solarbio. The commercial kit of serum Cr was from Beijing Leagene Biotechnology co. LTD.

## Results

### Anti-septic action of E_2_ by mitigating inflammatory cytokines release of septic mice

**Figure 1:**
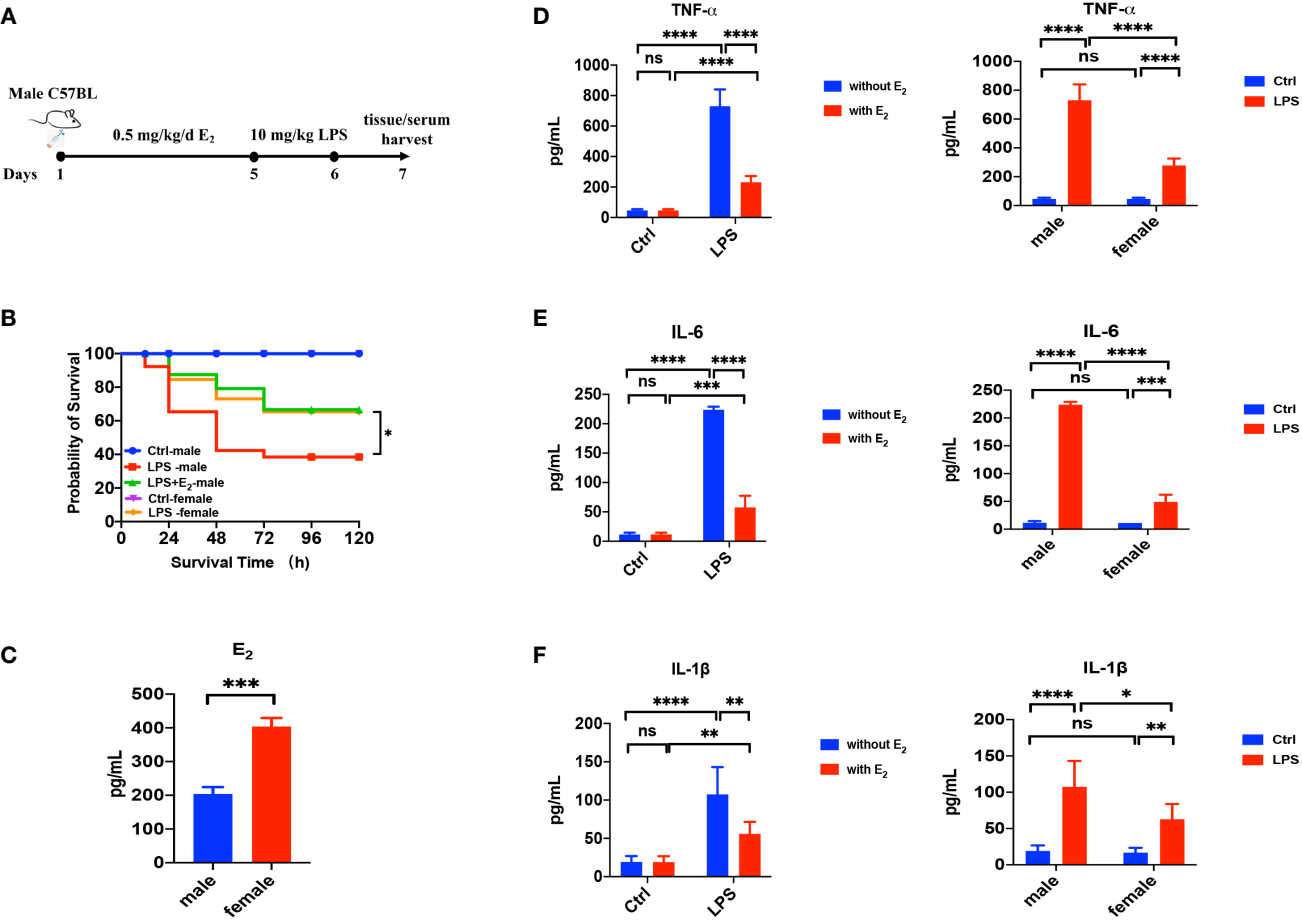
The beneficial effect of E_2_ on the survival rate and inflammatory factors in LPS-induced sepsis mice. **(A)** The experimental protocol of LPS-induced septic model of mice to study the action of E_2_ on sepsis. **(B)** Effect of E_2_ on the survival rate of septic mice in different experimental groups (n=25, the survival rate of the control group of female or male mice was 100%). **(C)** Generalize data shows serum estrogen of female mice and male mice (n=5). **(D–F)** Generalize data shows male mice serum TNF-α, IL-6 and IL-1β from control (ctrl), E_2_ group, LPS-induced sepsis group and LPS+E_2_ group (left panels, n=4). Comparison of serum TNF-α, IL-6 and IL-1β from the control group and LPS-induced sepsis group between female mice and male mice (right panels). *P <0.05, **P <0.01, ***P <0.001, ****P <0.0001 and ns, no significance.

First, we applied LPS-induced septic model of mice to study the action of E_2_ on sepsis, and our experimental protocol was shown in **Figure 1A**. The survival rate of LPS-induced sepsis was greater in female mice than in male mice, and E_2_ pretreatment (0.5 mg/kg/d subcutaneously for 5 days) significantly improved the survival rate of male septic mice (**Figure 1B**), indicating the anti-septic action of E_2_, which accords with an earlier observation. (24) Accordingly, E_2_ concentrations in female group were obviously higher than male group (**Figure 1C**). Second, we examined if the development of LPS-induced tissue injury was closely related to the enhanced inflammation. As shown in **Figures 1D–F** (left panels), serum levels of inflammatory cytokines, including TNF-α, IL-1β and IL-6, were significantly increased in the LPS group, while pretreatment with E_2_ effectively reduced the release of inflammatory cytokines. Moreover, LPS-induced inflammatory cytokines were significantly lower in female septic group compared to male septic group (right panels in **Figures 1D–F**). Therefore, E_2_ can mitigate inflammatory cytokines release to ameliorate septic survival rate.

### Protective effects of E_2_ on sepsis-induced multiple organ injuries

**Figure 2:**
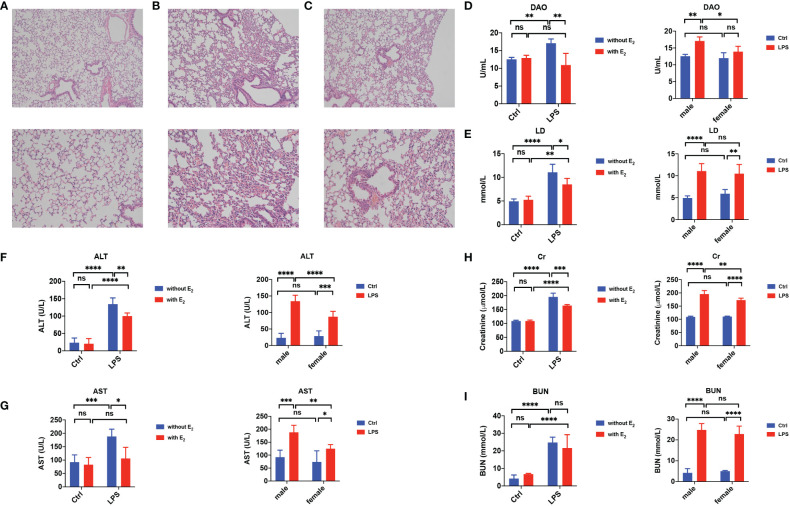
Protective effect of E_2_ in sepsis-induced tissue injury. **(A–C)** Hematoxylin-and-Eosin stain of the lung from healthy male mice **(A)**, LPS-induce male sepsis **(B)**, LPS+E_2_ male mice (C, n=5, magnification: × 100; × 200). **(D, E)** Summarized data shows serum diamine oxidase (DAO, D, n=5) and lactic acid (LD, E, n=5) from healthy male mice (Ctrl), E_2_ male mice, LPS-induce male sepsis, LPS+E_2_ male mice (left panels). Comparison of serum DAO and LD from the healthy group and LPS-induced sepsis group between female mice and male mice (right panels). **(F, G)** Summarized data shows serum alanine transaminase (ALT, F, n=5) and aspartate transaminase (AST, G, n=5) from healthy male mice (Ctrl), E_2_ male mice, LPS-induce male sepsis, LPS+E_2_ male mice (left panels). Comparison of serum ALT and AST from the healthy group and LPS-induced sepsis group between female mice and male mice (right panels). **(H, I)** Summarized data shows serum Creatinine (Cr, H, n=5) and blood urea nitrogen (BUN, I, n=5) from healthy male mice (Ctrl), E_2_ male mice, LPS-induce male sepsis, LPS+E_2_ male mice (left panels). Comparison of serum Cr and BUN from the healthy group and LPS-induced sepsis group between female mice and male mice (right panels). *P <0.05, **P <0.01, ***P <0.001, ****P <0.0001 and ns, no significance.

Since sepsis is a life-threatening disease with multiple organ failures including the lung, intestine, liver and kidney, we tested if E_2_ has a beneficial role in sepsis-induced tissue damage. As shown in H&E staining, compared to the normal lung (**Figure 2A**), obvious pathological changes in the lung were observed in the sepsis group, including abundant inflammatory cell infiltration and interstitial edema (**Figure 2B**). However, E_2_ pretreatment (0.5 mg/kg/d subcutaneously for 5 days) could markedly improve these pathological changes (**Figure 2C**).

It is acknowledged that serum lactate (LD), a product of intestinal bacteria glycolysis, and diamine oxidase (DAO), a high-activity intracellular enzyme in the intestinal villi that can be released into the blood from the damaged intestinal mucosa, are considered sensitive indicators for the intestinal mucosal barrier. Indeed, serum levels of both LD and DAO were increased in male septic mice compared to control mice, but E_2_ pretreatment significantly decreased their serum levels (left panels in **Figures 2D, E**). In addition, in the LPS-induced septic group, the serum level of DAO in females was lower than in males (right panels in **Figure 2D**).

Moreover, the serum levels of aspartate transaminase (AST) and alanine transaminase (ALT), important clinical parameters of hepatic function, were significantly elevated in male septic mice compared to control mice. At the same time, E_2_ pretreatment significantly decreased the LPS-induced elevation of these hepatic enzymes (left panels in **Figures 2F, G**). In septic mice, the serum levels of ALT and AST were lower in females than in males (right panels in **Figures 2F, G**). Finally, the serum levels of creatinine (Cr) and blood urea nitrogen (BUN), important clinical parameters of renal function, were significantly elevated in male septic mice (left panels in **Figures 2H, I**), and E_2_ pretreatment significantly decreased the LPS-induced elevation of Cr (left panels in **Figure 2H**). In septic mice, the serum level of Cr was lower in females than in males (right panels in **Figure 2H**). Therefore, E_2_ can protect against multiple organ dysfunction in sepsis.

### ERα mediated EDH-induced vasorelaxation *via* PLC/IP_3_ pathway

**Figure 3:**
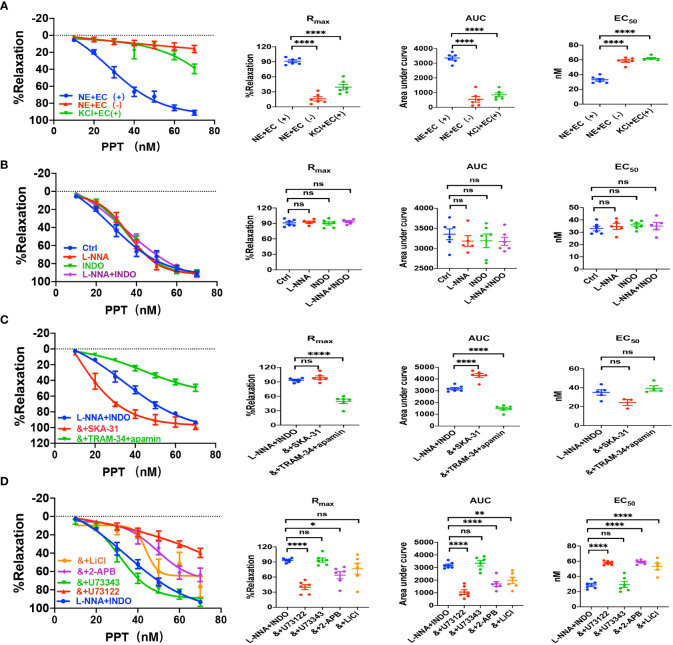
PPT induced EDH-mediated vasorelaxation of the second-order branch of mouse mesenteric arterioles *via* PLC/IP_3._ **(A)** Summarized data shows PPT induced a concentration-dependent relaxation response of mouse mesenteric arterioles with endothelium-intact (EC+, n=6) or endothelium-denuded (EC-, n=6) pre-constricted with noradrenalin (NE, 5 μM) or high K^+^ (KCl, 80 mM, n=6) in the CRC, R_max_, AUC and EC_50_. **(B)** Summarized data shows PPT-induced CRC, R_max_, AUC and EC_50_ of mouse mesenteric arterioles pre-constricted with NE without (ctrl, n=6), or with either 100 μM L-NNA (n=6), or 10 μM INDO (n=6), or 100 μM L-NNA plus 10 μM INDO (n=6). **(C)** Summarized data shows PPT-induced CRC, R_max_, AUC and EC_50_ of mouse mesenteric arterioles pre-constricted with NE with either L-NNA plus INDO (n=6), L-NNA + INDO (&) + 0.3 μM SKA-31 (n=6), or L-NNA + INDO (&) + 3 μM apamin + 30 μM TRAM-34 (n=6). **(D)** Summarized data shows PPT-induced CRC, R_max_, AUC and EC_50_ of mouse mesenteric arterioles pre-constricted with NE with either L-NNA plus INDO (n=6), L-NNA + INDO (&) + 10 μM U73122 (n=6), L-NNA + INDO (&) + 10 μM U73343 (n=6), L-NNA + INDO (&) + 40 μM 2-APB (n=6), or L-NNA + INDO (&) + 30 mM LiCl (n=6). Data are expressed as a percentage of 5 μM NE or 80 mM KCl induced contractions and expressed as means ± SEM. *P <0.05, **P <0.01, ****P <0.0001 and ns, no significance.

Sepsis is usually accompanied by microvascular dysfunction, finally resulting in hypoperfusion and multiple organ failure. Although E_2_ can exert beneficial effects on sepsis-induced multiple organ dysfunction, it is unknown if vascular ER subtypes are involved in these benefits in sepsis. Therefore, we applied PPT, a selective ERα activator, to examine vascular action of ERα and the underlying mechanisms. As shown in **Figure 3A**, PPT dose-dependently induced vasorelaxation of mouse mesenteric arterioles, which was mostly endothelium-dependent. Moreover, PPT hardly induced vasorelaxation of the arterioles pre-constricted by high K^+^ (80 mM), and there were significant differences in PPT-induced CRC, R_max_, AUC, and EC_50_ between the arterioles pre-constricted by NE and high K^+^, suggesting a possible involvement of cellular K^+^ gradients.

Since VEC mediates vasorelaxation via NO, PGI_2_, and EDH, we examined their contributions to PPT-induced vasorelaxation. As shown in **Figure 3B**, either L-NNA (100 μM), a selective inhibitor of NO, or INDO (10 μM), a selective inhibitor of PGI_2_, did not affect PPT-induced CRC, R_max_, AUC, and EC_50_ in comparison to the control. Furthermore, PPT-induced vasorelaxation was barely affected by L-NNA plus INDO (**Figure 3B**), indicating minor roles of NO and PGI_2_ in PPT-induced vasorelaxation. However, when L-NNA and INDO eliminated NO and PGI_2_, PPT-induced vasorelaxation was significantly inhibited by apamin (3 μM) plus TRAM-34 (30 μM), selective blockers of SK_Ca_ and IK_Ca_; but potentiated by SKA-31 (0.3 μM), a selective activator of SK_Ca_ and IK_Ca_ (**Figure 3C**), indicating that EDH plays a predominant role in PPT-induced vasorelaxation.

Since EDH is regulated not only by endothelial IK_Ca_ and SK_Ca_, but also by Na^+^-K^+^ ATPase (NKA) and Na^+^/Ca^2+^ exchanger (NCX) on VSMCs, we further examined if NKA and NCX are involved in PPT-induced vasorelaxation. Indeed, in the presence of L-NNA and INDO, PPT-induced vasorelaxation was significantly attenuated by ouabain (100 μM), a selective inhibitor of NKA, and SN-6 (10 μM), a selective inhibitor of NCX (**Supplementary Figure 1A**). Finally, to confirm the role of ERα in PPT-induced vasorelaxation, we applied MPP (1 μM), a selective inhibitor of ERα. As shown in **Supplementary Figure 1A**, PPT-induced vasorelaxation was significantly inhibited by MPP. Therefore, ERα subtype mediates vasorelaxation of mesenteric arterioles via EDH mechanism.

Due to the critical role of PLC/IP_3_ pathway in the process of membrane receptor activation, we further examined its possible involvement in PPT-induced vasorelaxation. First, in the presence of L-NNA and INDO, PPT-induced vasorelaxation was significantly attenuated by U73122 (10 μM), a selective PLC inhibitor but was unaffected by U73343 (10 μM), an inactive analog of U-73122 (**Figure 3D**). Second, PPT-induced vasorelaxation was inhibited by LiCl (30 mM), an inhibitor of IP_3_ production, and 2-APB (40 μM), an inhibitor of IP_3_ receptor (IP_3_R) (**Figure 3D**). 2-APB can not only inhibit IP_3_R in vascular endothelial cells, leading to a decrease in Ca^2+^ signaling to attenuate endothelium-dependent vasorelaxation, but it can also activate some TRP channels to cause an increase in Ca^2+^ signaling and to promote vasorelaxation. (25–27) Therefore, in the present study, PPT-induced vasorelaxation was significantly inhibited by 2-APB, indicating it inhibits IP_3_R rather than activates TRP channels. Taken together, ERα subtype regulates EDH-mediated vasorelaxation *via* PLC/IP_3_ pathway.

### ERβ- and GPER-mediated EDH-induced vasorelaxation *via* PLC/IP_3_ pathway

**Figure 4:**
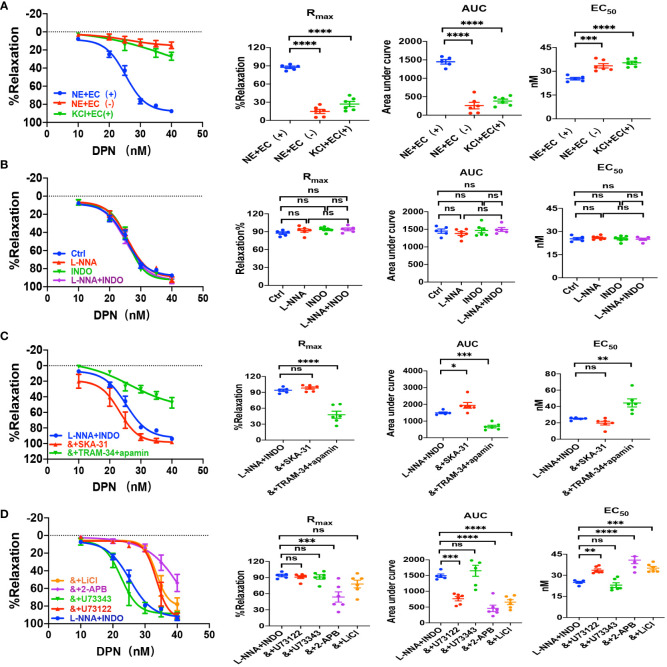
DPN induced EDH-mediated vasorelaxation of the second-order branch of mouse mesenteric arterioles *via* PLC/IP_3_. **(A)** Summarized data shows DPN induced a concentration-dependent relaxation response of mouse mesenteric arterioles with endothelium-intact (EC+, n=6) or endothelium-denuded (EC-, n=6) pre-constricted with noradrenalin (NE, 5 μM) or high K^+^ (KCl, 80 mM, n=6) in the CRC, R_max_, AUC and EC_50_. **(B)** Summarized data shows DPN-induced CRC, R_max_, AUC and EC_50_ of mouse mesenteric arterioles pre-constricted with NE without (ctrl, n=6), or with either 100 μM L-NNA (n=6), or 10 μM INDO (n=6), or 100 μM L-NNA plus 10 μM INDO (n=6). **(C)** Summarized data shows DPN-induced CRC, R_max_, AUC and EC_50_ of mouse mesenteric arterioles pre-constricted with NE with either L-NNA plus INDO (n=6), L-NNA + INDO (&) + 0.3 μM SKA-31 (n=6), or L-NNA + INDO (&) + 3 μM apamin + 30 μM TRAM-34 (n=6). **(D)** Summarized data shows DPN-induced CRC, R_max_, AUC and EC_50_ of mouse mesenteric arterioles pre-constricted with NE with either L-NNA plus INDO (n=6), L-NNA + INDO (&) + 10 μM U73122 (n=6), L-NNA + INDO (&) + 10 μM U73343 (n=6), L-NNA + INDO (&) + 40 μM 2-APB (n=6), or L-NNA + INDO (&) + 30 mM LiCl (n=6). Data are expressed as a percentage of 5 μM NE or 80 mM KCl induced contractions and expressed as means ± SEM. *P <0.05, **P <0.01, ***P <0.001, ****P <0.0001 and ns, no significance.

**Figure 5:**
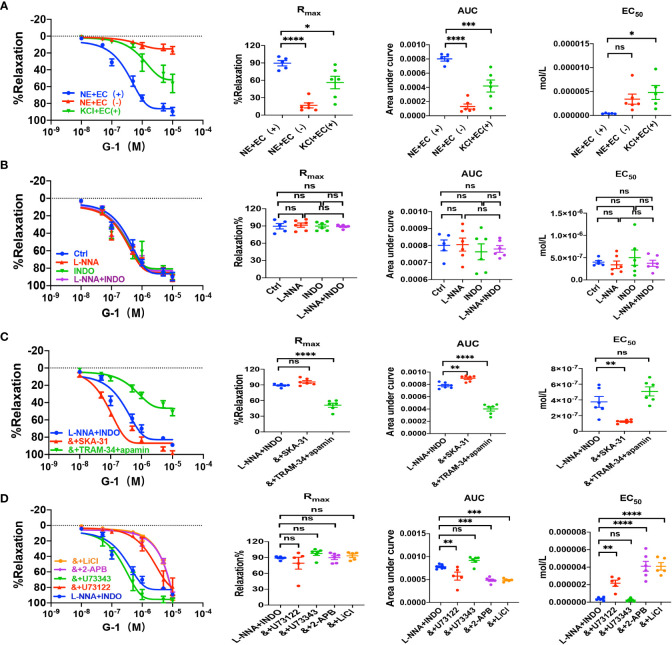
G-1 induced EDH-mediated vasorelaxation of the second-order branch of mouse mesenteric arterioles *via* PLC/IP_3_. **(A)** Summarized data shows G-1 induced a concentration-dependent relaxation response of mouse mesenteric arterioles with endothelium-intact (EC+, n=6) or endothelium-denuded (EC-, n=6) pre-constricted with noradrenalin (NE, 5 μM) or high K^+^ (KCl, 80 mM, n=6) in the CRC, R_max_, AUC and EC_50_. **(B)** Summarized data shows G-1-induced CRC, R_max_, AUC and EC_50_ of mouse mesenteric arterioles pre-constricted with NE without (ctrl, n=6), or with either 100 μM L-NNA (n=6), or 10 μM INDO (n=6), or 100 μM L-NNA plus 10 μM INDO (n=6). **(C)** Summarized data shows G-1-induced CRC, R_max_, AUC and EC_50_ of mouse mesenteric arterioles pre-constricted with NE with either L-NNA plus INDO (n=6), L-NNA + INDO (&) + 0.3 μM SKA-31 (n=6), or L-NNA + INDO (&) + 3 μM apamin + 30 μM TRAM-34 (n=6). **(D)** Summarized data shows G-1-induced CRC, R_max_, AUC and EC_50_ of mouse mesenteric arterioles pre-constricted with NE with either L-NNA plus INDO (n=6), L-NNA + INDO (&) + 10 μM U73122 (n=6), L-NNA + INDO (&) + 10 μM U73343 (n=6), L-NNA + INDO (&) + 40 μM 2-APB (n=6), or L-NNA + INDO (&) + 30 mM LiCl (n=6). Data are expressed as a percentage of 5 μM NE or 80 mM KCl induced contractions and expressed as means ± SEM. *P <0.05, **P <0.01, ***P <0.001, ****P <0.0001 and ns, no significance.

We applied either DPN, a selective ERβ activator, or G-1, a selective GPER activator, to examine vascular action of ERβ and EPER and the underlying mechanisms. As shown in **Figures 4A** and **5A**, either DPN or G-1 induced vasorelaxation in dose-dependent and endothelium-dependent manners. There were significant differences in DPN- and GPER-induced CRC, R_max_, AUC, and EC_50_ between the arterioles pre-constricted by NE and high K^+^ (**Figures 4A**, **5A**).

As shown in **Figures 4B** and **5B**, L-NNA and INDO have no effects on DPN- and G-1-induced CRC, R_max_, AUC, and EC_50_ compared to their controls. However, in the presence of L-NNA plus INDO, DPN- and G-1-induced vasorelaxation was inhibited by apamin plus TRAM-34; but potentiated by SKA-31 (**Figures 4C**, **5C**), further suggesting that DPN- and GPER-mediated vasorelaxation via EDH predominantly. As shown in **Supplementary Figures 1B, C**, in the presence of L-NNA and INDO, DPN- and G-1-induced vasorelaxation was significantly attenuated by ouabain and SN-6 respectively, indicating the involvements of NKA and NCX. Finally, we applied either PHTPP, a selective inhibitor of ERβ, or G-15, a selective inhibitor of GPER, to examine the involvements of ERβ and GPER. As shown in **Supplementary Figures 1B, C**, in the presence of PHTPP (3 μM) and G-15 (1 μM), DPN- and G-1-induced vasorelaxation was significantly attenuated, indicating ERβ and GPER subtypes mediate vasorelaxation of mesenteric arterioles via EDH mechanism.

We also examined the possible involvements of PLC/IP_3_ pathway in ERβ- and GPER-induced vasorelaxation. As shown in **Figures 4D**, **5D**, in the presence of L-NNA and INDO, DPN- and G-1-induced vasorelaxation was significantly attenuated by U73122, LiCl and 2-APB, but was unaffected by U73343. Taken together, ERβ- and GPER-mediated EDH-induced vasorelaxation via PLC/IP_3_ pathway.

### ER subtype-induced Ca^2+^ signaling in VEC via PLC/IP_3_/IP_3_R pathway

**Figure 6:**
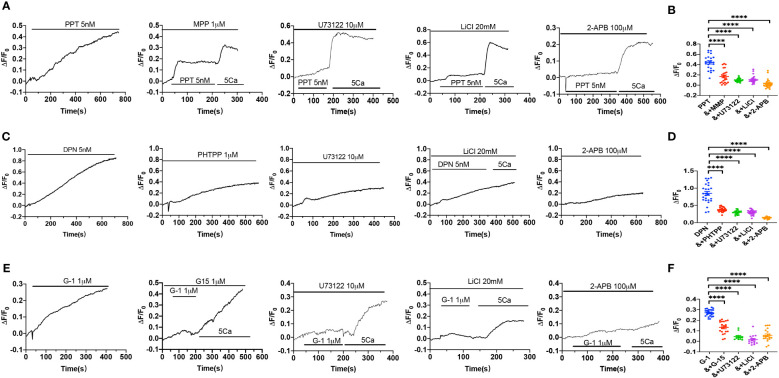
Activation of estrogen receptors induced Ca^2+^ signal *via* PLC/IP_3_/IP_3_R pathway. **(A)** Summary data shows the time courses of PPT (5 nM)-induced Ca^2+^ signaling in the absence (n=26 cells) or the presence of MPP (1 μM, n=23 cells), U73122 (10 μM, n=22 cells), LiCl (20 mM, n=22 cells) or 2-APB (100 μM, n=20 cells) in Ca^2+^-containing solutions. **(B)** Summary data shows the peaks of PPT-increased [Ca^2+^]_i_ signaling in the absence or the presence of MPP, U73122, LiCl, or 2-APB in Ca^2+^-containing solutions. **(C)** Summary data shows the time courses of DPN (5 nM)-induced Ca^2+^ signaling in the absence (n=23 cells) or the presence of PHTPP (1 μM, n=24 cells), U73122 (10 μM, n=24 cells), LiCl (20 mM, n=23 cells) or 2-APB (100 μM, n=28 cells) in Ca^2+^-containing solutions. **(D)** Summary data shows the peaks of DPN-increased [Ca^2+^]_i_ signaling in the absence or the presence of PHTPP, U73122, LiCl, or 2-APB in Ca^2+^-containing solutions. **(E)** Summary data shows the time courses of G-1 (1 μM)-induced Ca^2+^ signaling in the absence (n=25 cells) or the presence of G-15 (1 μM, n=21 cells), U73122 (10 μM, n=12 cells), LiCl (20 mM, n=15 cells) or 2-APB (100 μM, n=19 cells) in Ca^2+^-containing solutions. **(F)** Summary data shows the peaks of G-1-increased [Ca^2+^]_i_ signaling in the absence or the presence of G-15, U73122, LiCl, or 2-APB in Ca^2+^-containing solutions. Data are shown as means of fluorescence ratio (ΔF/F0) in every single cell. ****P <0.0001.

We further verified if ER subtypes induce Ca^2+^ signaling in HUVEC via PLC/IP_3_/IP_3_R pathway. As shown in **Figures 6A, B**, PPT-induced obvious [Ca^2+^]_cyt_ signaling, which was attenuated by MPP, U73122, LiCl and 2-APB, indicating that PPT-induced endothelial [Ca^2+^]_cyt_ signaling via ERα/PLC/IP_3_/IP_3_R pathway. Similarly, DPN-induced [Ca^2+^]_cyt_ signaling was attenuated by PHTPP, U73122, LiCl and 2-APB, indicating that DPN-induced endothelial [Ca^2+^]_cyt_ signaling via ERβ/PLC/IP_3_/IP_3_R pathway (**Figures 6C, D**). Finally, G-1-induced [Ca^2+^]_cyt_ signaling was attenuated by G-15, U73122, LiCl and 2-APB, indicating that G-1 induced endothelial [Ca^2+^]_cyt_ signaling via GPER/PLC/IP_3_/IP_3_R pathway (**Figures 6E, F**). Therefore, these data further support that endothelial PLC/IP_3_/IP_3_R pathway plays an important role in ER subtype-induced vasorelaxation.

### Protective role of E_2_/ER-mediated vasorelaxation against sepsis

**Figure 7:**
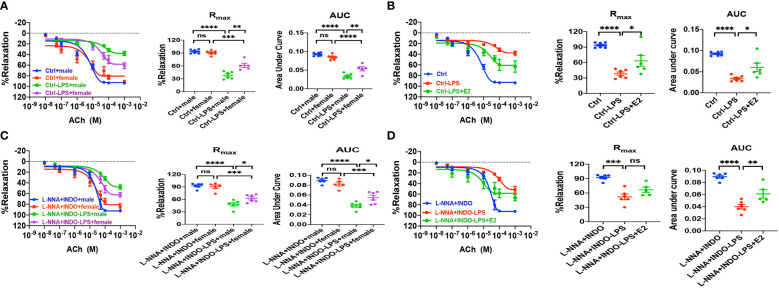
In LPS-induced sepsis mice, E_2_ can contribute to gender differences in vasorelaxation dysfunction. **(A, C)** Summary data shows acetylcholine (ACh)-induced CRC, R_max_, and AUC of male or female mice after different treatments without (control) or with LPS intraperitoneal injection in the absence/presence of 100 μM L-NNA plus 10 μM INDO (n=6). **(B, D)** Summary data shows acetylcholine (ACh)-induced CRC, R_max_, and AUC of male mice after different treatments without (control) or with LPS intraperitoneal injection or LPS and E_2_ pretreatment in the absence/presence of L-NNA plus INDO (n=6). Data are expressed as a percentage of NE (5 μM)-induced contractions and expressed as means ± SEM. *P <0.05, **P <0.01, ***P <0.001, ****P <0.0001 and ns, no significance.

As described earlier, we found the survival rate of sepsis was greater in female mice than in male mice due to the protective effect of E_2_ *via* ER activation; however, nothing is known if ER subtype-mediated vasorelaxation contributes to the beneficial effect of E_2_. To this end, first, we examined the relaxation response to vagus neurotransmitter acetylcholine (ACh), an acknowledged vasodilator, in LPS-induced septic mice. As shown in **Figure 7A**, there were no differences in ACh-induced vasorelaxation between healthy male and female mice. However, in LPS-induced septic mice, ACh-induced vasorelaxation was more impaired in males than in females. Furthermore, in the presence of L-NNA and INDO, there were no differences in ACh-induced vasorelaxation between male and female mice. However, in LPS-induced septic mice, ACh-induced vasorelaxation was more impaired in males than in females (**Figure 7B**). Finally, in LPS-induced male septic mice, ACh-induced vasorelaxation was significantly impaired, but E_2_ (0.5 mg/kg/d subcutaneously for 5 days) pretreatment rescued the impaired ACh-induced EDH-mediated vasorelaxation (**Figures 7C, D**).

**Figure 8:**
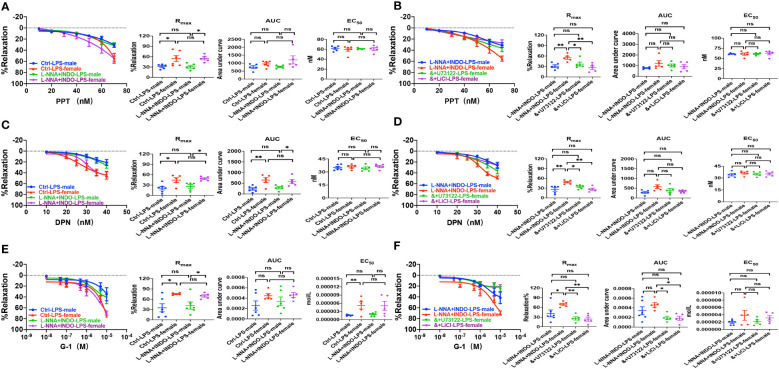
PLC/IP_3_ pathway contributed to gender differences in activation of estrogen receptors-induced vasorelaxation in LPS-induced sepsis mice. **(A)** Summary data shows PPT-induced CRC, R_max_, AUC and EC_50_ of male or female mice after treatment with LPS intraperitoneal injection in the absence/presence of 100 μM L-NNA plus 10 μM INDO (n=6). **(B)** Summary data shows PPT-induced CRC, R_max_, AUC and EC_50_ of male or female mice after treatment with LPS intraperitoneal injection with L-NNA plus INDO (n=6), L-NNA + INDO (&) + 10 μM U73122 (n=6), or L-NNA + INDO (&) + 30 mM LiCl (n=6). **(C)** Summary data shows DPN-induced CRC, R_max_, AUC and EC_50_ of male or female mice after treatment with LPS intraperitoneal injection in the absence/presence of L-NNA plus INDO (n=6). **(D)** Summary data shows DPN-induced CRC, R_max_, AUC and EC_50_ of male or female mice after treatment with LPS intraperitoneal injection with L-NNA plus INDO (n=6), L-NNA + INDO (&) + 10 μM U73122 (n=6), or L-NNA + INDO (&) + 30 mM LiCl (n=6). **(E)** Summary data shows G-1-induced CRC, R_max_, AUC and EC_50_ of male or female mice after treatment with LPS intraperitoneal injection in the absence/presence of L-NNA plus INDO (n=6). **(F)** Summary data shows G-1-induced CRC, R_max_, AUC and EC_50_ of male or female mice after treatment with LPS intraperitoneal injection with L-NNA plus INDO (n=6), L-NNA + INDO (&) + 10 μM U73122 (n=6), or L-NNA + INDO (&) + 30 mM LiCl (n=6). Data are expressed as a percentage of 5 μM NE induced contractions and expressed as means ± SEM. *P <0.05, **P <0.01 and ns, no significance.

Second, we examined if ER subtype-mediated vasorelaxation plays a protective role against sepsis. As shown in **Figures 8A, C, E**, in the absence or the presence of L-NNA and INDO, PPT-, DPN- and G-1-induced vasorelaxation was more impaired in septic male mice than female mice. Taken together, septic female mice are more resistant to the dysfunction in EDH-mediated vasorelaxation because ER subtypes play protective roles against sepsis.

### Contribution of PLC/IP_3_ pathway to the gender difference in EDH-mediated vasorelaxation in sepsis

Although we found that PLC/IP_3_ pathway is involved in ER-induced vasorelaxation under physiological situations, nothing is known about its contribution to the gender differences in EDH-mediated vasorelaxation in sepsis. As shown in **Figures 8B, D, F**, in the presence of L-NNA and INDO, the EDH-mediated vasorelaxation induced by PPT, DPN, and G-1 was inhibited by U73122 or LiCl in septic female mice, leading to the abolishment of the gender differences in PPT-, DPN- and G-1-induced EDH-mediated vasorelaxation between septic female and male mice. Taken together, PLC/IP_3_ pathway contributes to the gender differences in EDH-mediated vasorelaxation in sepsis.

### Protective role of endothelial E_2_/ER against sepsis *via* PLC/IP_3_R pathway

Since sepsis is usually accompanied by VEC dysfunction, and proinflammatory cytokines IL-1β, IL-6 and TNF-α are important in the occurrence and development of sepsis, we first examined if E_2_ can affect release of inflammatory cytokines from HUVEC. As shown in **Supplementary Figures 2A, B**, compared to ctrl group, both mRNA expression and release of IL-1β, IL-6 and TNF-α were significantly enhanced in LPS-pretreated group, which were reduced by pretreatment with E_2_ (10 nM).

We further examined the contributions of ER subtypes to E_2_-mediated inflammatory responses, As shown in **Supplementary Figures 3A-C**, the release of proinflammatory cytokines IL-1β, IL-6 and TNF-α from HUVEC were increased by LPS, which were attenuated by PPT, DPN, and G-1, respectively. Taken together, E_2_ can activate ER subtypes to exert protective effects by inhibiting release of inflammatory cytokines from VEC in sepsis. Finally, we examined if endothelial PLC/IP_3_R pathway is involved in the protective effect of E_2_ against sepsis. As shown in **Supplementary Figures 4A–C**, compared to ctrl group, both mRNA and protein expressions of PLC and IP_3_R in HUVEC were significantly increased in LPS-pretreated group, which could be rescued by E_2_. These data further support that E_2_ exerts protective effect against sepsis *via* endothelial PLC/IP_3_R pathway.

## Discussion

Since sepsis is a life-threatening disease to cause high mortality worldwide, it is pivotal to elucidate its pathogenesis and to seek for effective therapeutic targets. In the present study, we investigated the actions and mechanisms of E_2_/ER subtypes in protection against sepsis from the view of arterioles. The major novel findings of this study are: (1) E_2_ sex-dependently improves the survival rate and inhibits proinflammatory cytokines in septic male mice; (2) E_2_ ameliorates pulmonary, intestinal, hepatic and renal multiple organ injuries in septic male mice; (3) ER subtypes inhibit proinflammatory cytokines in VEC via PLC/IP_3_/IP_3_R pathway; (4) ER subtypes mediate EDH-induced vasorelaxation via PLC/IP_3_ pathway, which was more impaired in septic male than female mice; (5) E_2_/ER subtypes can rescue the impaired ACh-induced EDH-mediated vasorelaxation in septic male mice via PLC/IP_3_ pathway.

Several clinical epidemiological studies reported that females are less susceptible to posttraumatic infections and multiple organ failures. (4) Recent studies reveal that the phenomenon might be caused mainly by sex hormones. (5, 8) It was found that in sepsis, estrogens can exert anti-oxidant and anti-inflammatory properties in immune cells, while androgens have harmful opposite effects. In the present study, LPS-induced septic female mice had higher survival rate compared to septic male mice, and E_2_ pretreatment significantly improved the survival rate of septic male mice to abolish the sex differences. Moreover, E_2_ attenuated the elevated levels of serum proinflammatory cytokines IL-1β and IL-6 and TNF-α in septic male mice. Similarly, LPS-induced release of TNF-α, IL-1β and IL-6 from VEC was attenuated by E_2_ and selective activators of three ER subtypes. Therefore, we not only verify sex differences in sepsis, but also provide insights into the protective roles of endothelial E_2_/ER subtypes.

As sepsis progresses, multiple organ dysfunction occurs, resulting in high mortality in sepsis. Therefore, it is critical to protect against septic multiple organ failures in clinics. We found that LPS-induced septic female mice had minor multiple organ dysfunction compared to septic male mice, Moreover, E_2_ can not only ameliorate multiple organ dysfunction induced by sepsis, but also alleviate organic damage, further supporting beneficial role of E_2_ in sepsis.

**Figure 9:**
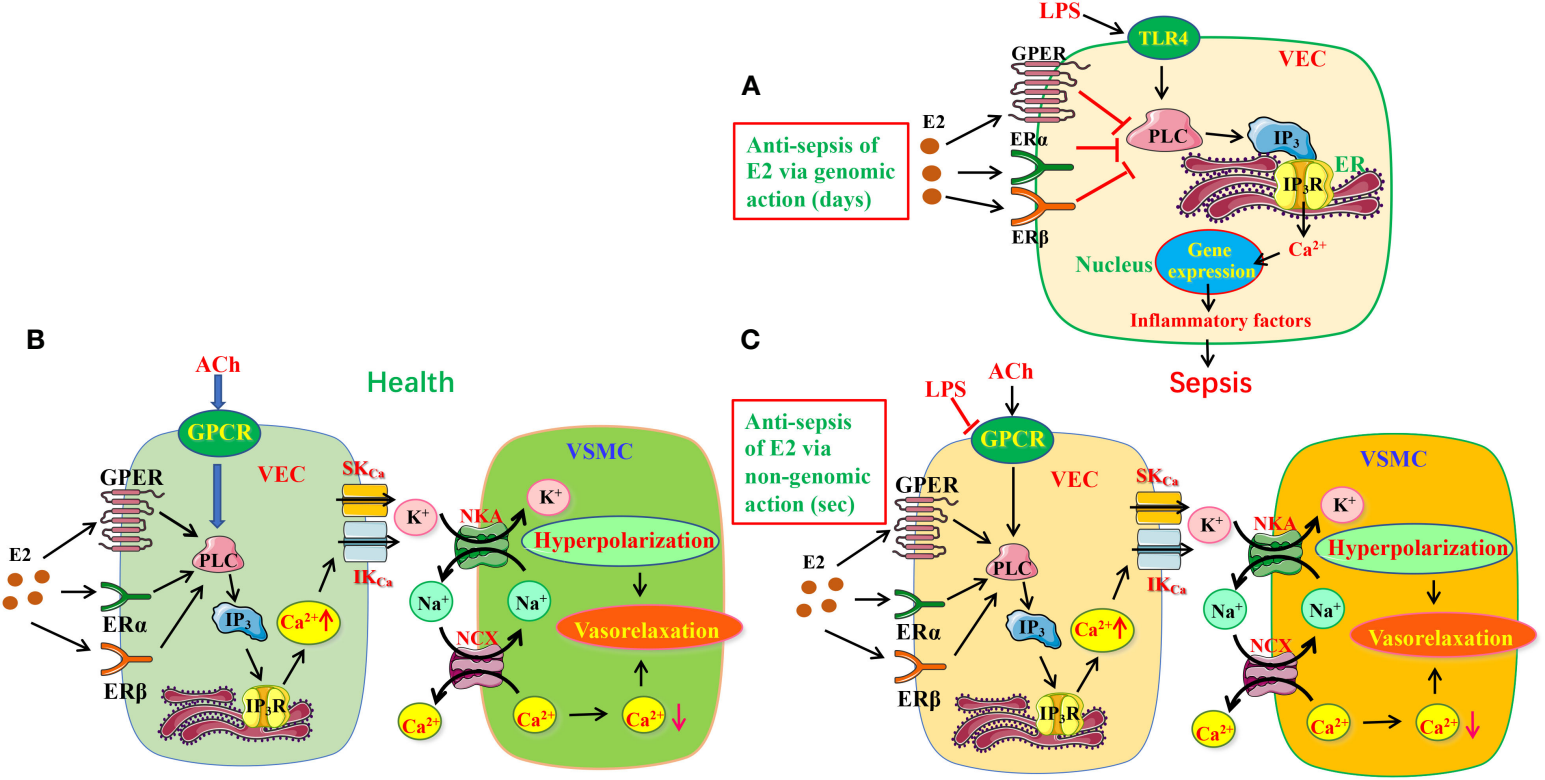
The protective mechanisms of estrogen/estrogen receptors in LPS-induced sepsis models. **(A)** The schematic diagram depicting estrogen/ER can modulate PLC/IP_3_R pathway to exert anti-inflammation *via* genomic action. **(B, C)** Estrogen/ER induced EDH-mediated vasorelaxation in health **(B)** and its protective effects in sepsis **(C)**. Estrogen/ER can modulate PLC/IP_3_R pathway to increase the release of ER Ca^2+^ *via* non-genomic action. Increased Ca^2+^ signaling stimulates IK_Ca_ and SK_Ca_ on VEC, leading to K^+^ efflux activating NKA, further inducing vasorelaxation through hyperpolarization. Moreover, NKA activation reduces [Na^+^]_i_ in VSMC, which stimulates NCX activity to decrease [Ca^2+^]_i_, resulting in further vasorelaxation. While the ACh/GPCR/EDH-mediated vasodilation is significantly impaired in sepsis, estrogen/ER-induced endothelium-dependent vasorelaxation takes over. TLR4, Toll-like receptor 4; VEC, vascular endothelial cells; VSMC, vascular smooth muscle cells; ER, endoplasmic reticulum; IK_Ca_ and SK_Ca_, intermediate and small conductance of Ca^2+^-activated K^+^ channels; NKA, Na^+^/K^+^-ATPase; NCX, Na^+^/Ca^2+^-exchanger; ER, estrogen receptors.

It is currently acknowledged that LPS activates Toll-like receptor 4 (TLR4) to initiate proinflammatory cascades, in which large amounts of cytokines are released *via* several signaling pathways, including mitogen-activated protein kinase (MAPK) cascade, phosphoinositide-specific phospholipase C (PLC)/protein kinase C (PKC)/phosphatidylinositol 3-kinase (PI3K), and Src/Akt pathway to prompt disease development. (28, 29) However, in the present study, we provide evidences to support that E_2_/ER subtypes inhibit endothelial PLC/IP_3_/Ca^2+^ signaling pathway *via* their genomic actions (for 7 days) to reduce proinflammatory cytokines, leading to anti-sepsis (**Figure 9A**).

Microcirculatory dysfunction, including endothelial damage, impaired vasomotion, and coagulation activation, may present as capillary leakage, hypotension, micro-thrombosis, and impaired tissue perfusion. Sepsis is usually accompanied by microcirculation dysfunction, leading to multiple organ injuries. (30, 31). Therefore, it is of great clinical significance to protect the microcirculation of tissues for sepsis therapy. VEC can produce three relaxing factors NO, PGI_2_ and EDH to induce vasorelaxation. (32, 33) EDH is known as a non-NO and non-PGI_2_ endothelium-dependent hyperpolarization. Although both NO and EDH are major vasodilators, the former is dominant in conduit arteries, but the latter is critical in resistance vessels. Importantly, when endothelial dysfunction results in reduced generation of NO, EDH can compensate to maintain the endothelium-dependent vasorelaxation of resistance vessels, indicating the importance of EDH in resistance vessels. (34, 35) Although the importance of NO in E_2_-induced vasorelaxation is well known, (36, 37) much uncertainty still exists about the relationship between ER and EDH-induced vasorelaxation. There are three ER subtypes: ERα, ERβ and GPER, but little is known about which subtypes involve in E_2_-induced vasorelaxation. Our study indicates that ERα, ERβ and GPER predominately induce EDH-mediated vasorelaxation of mesenteric arterioles via nongenomic action (for several seconds) in health (**Figure 9B**) because: 1) ER subtype activation induced a major endothelium-dependent vasorelaxation, which was much greater in the arterioles pre-constricted by NE than by high K^+^, 2) while ER-mediated vasorelaxation was unaffected by inhibitors of NO and PGI_2_, it was significantly attenuated by the inhibitors of IK_Ca_ and SK_Ca_ channels, but was potentiated by their activator; 3) ER-mediated vasorelaxation was attenuated by inhibitors of NCX and NKA; 4) ER-mediated vasorelaxation was largely attenuated by the selective inhibitors of ER subtypes.

Although ER can modulate multiple physiological activities via PLC/IP_3_/IP_3_R pathway, (38, 39) little is known about its involvement in ER-mediated vasorelaxation. In the present study, we reveal that PLC/IP_3_/IP_3_R/Ca^2+^ pathway plays a pivotal role in ER-mediated vasorelaxation because: 1) the vasorelaxation was attenuated by selective inhibitors of PLC/IP_3_/IP_3_R, but unaffected by an inactive analog of PLC inhibitor; 2) ER-induced Ca^2+^ signaling was inhibited by selective inhibitors of PLC/IP_3_/IP_3_R.

It was previously reported that E_2_ could exert beneficial effects by anti-oxidant and anti-inflammatory in sepsis. However, to our knowledge, it has not been explored if E_2_ can activate endothelial ERα/ERβ/GPER to induce vasorelaxation to finally protect against septic organ dysfunction. Not only has the present study certified that ERα, ERβ and GPER induce EDH-mediated vasorelaxation via PLC/IP_3_/IP_3_R pathway, but in sepsis when ACh-induced vasorelaxation was impaired, E_2_ pretreatment could rescue it to increase blood flow to the septic organs via nongenomic action-mediated PLC/IP_3_/IP_3_R pathway to finally exert beneficial effects (**Figure 9C**).

In conclusion, we not only reveal that E_2_/ER subtypes mediate anti-inflammation and vasorelaxation via genomic and nongenomic actions in septic mice, but also elucidate that endothelial ER subtypes reduce proinflammatory cytokines and induce EDH-mediated vasorelaxation via PLC/IP_3_R/Ca^2+^ pathway, finally ameliorating sepsis-induced organ injury and survival rate. Although more studies are needed for clarification in humans, we offer for the first time the actions and mechanisms of endothelial ER subtypes in microvascular function and in sepsis. Therefore, E_2_ has protective actions against sepsis since endothelial ER subtypes and PLC/IP_3_/IP_3_R are novel potential targets. However, our study has some limitations which need further investigations. For example, although both cecal ligation and puncture (CLP) and lipopolysaccharide are well-recognized sepsis models, it should be double checked with the CLP-induced sepsis model. In addition, to examine the precise actions of ER subtypes in endothelial cells, it would be the best to apply primary vascular endothelial cells obtained from mesenteric artery.

## Data availability statement

The original contributions presented in the study are included in the article/**Supplementary Material**. Further inquiries can be directed to the corresponding authors.

## Ethics statement

The animal study was reviewed and approved by Children’s Hospital of Chongqing Medical University.

## Author contributions

HD conceived the study, designed all the experiments, wrote and finalized the manuscript. FX designed some experiments. LZ, WL, MZ, and HW performed the experiments and data analysis. All authors contributed to the article and approved the submitted version.
