# Supplementary material for: Estrogen receptor subtype mediated anti-inflammation and vasorelaxation via genomic and nongenomic actions in septic mice
Source: Front Endocrinol (Lausanne). 2023 May 17;14:1152634. doi: 10.3389/fendo.2023.1152634 (PMC10230057; doi:10.3389/fendo.2023.1152634)
Supplement: Supplementary file 1 [file DataSheet_1.docx]

**Supplementary materials for “Estrogen receptor subtype-mediated anti-inflammation and vasorelaxation via genomic and nongenomic actions in septic mice”**

**
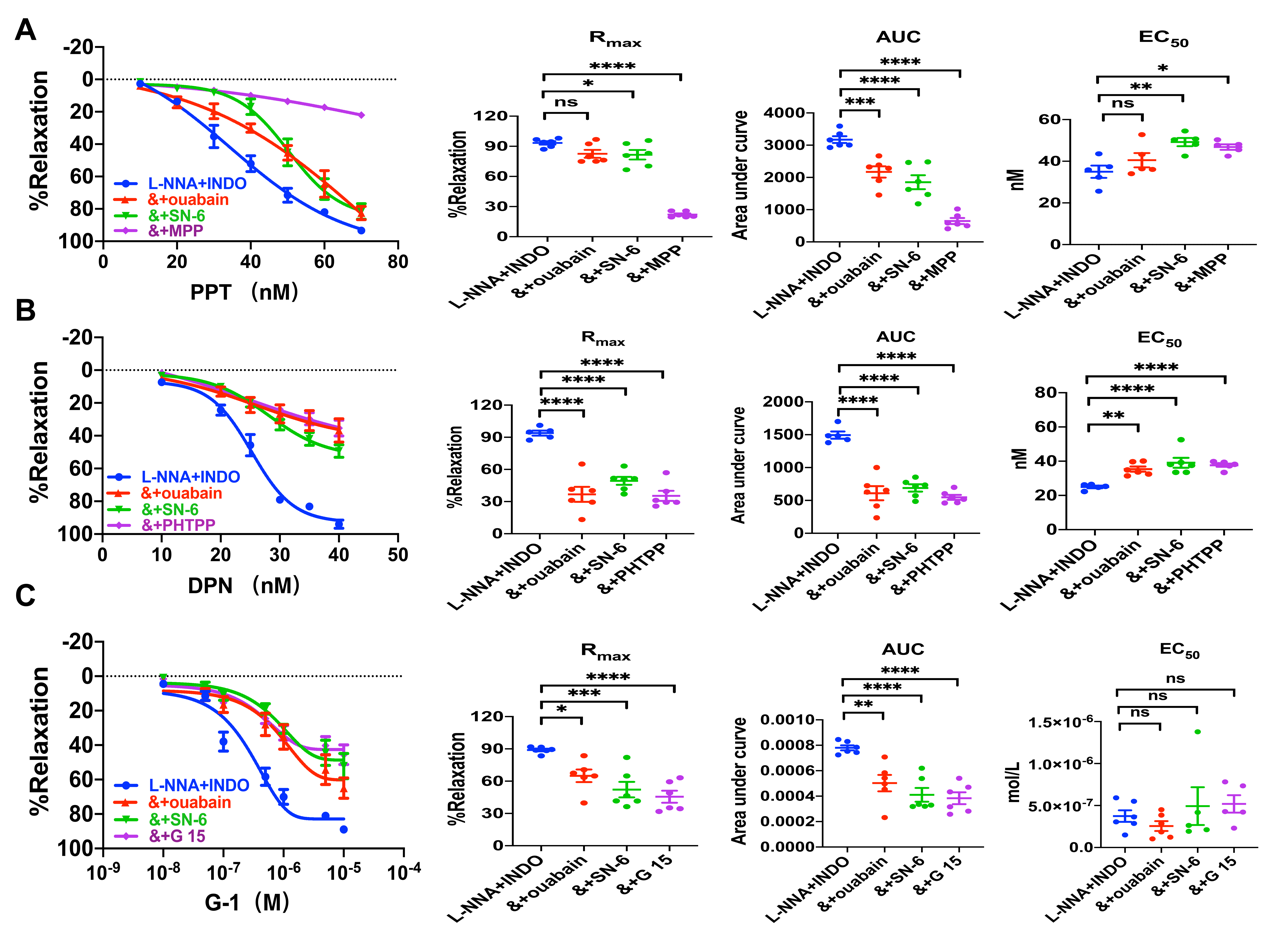
**

**Sup Figure 1. The involvement of Na^+^-K^+^ ATPase (NKA) and Na^+^/Ca^2+^ exchanger (NCX)** **in activation of estrogen receptors-induced vasorelaxation**

**(A)** Summarized data shows PPT-induced CRC, R_max_, AUC and EC_50_ of male mouse mesenteric arterioles pre-constricted by NE with 100 μM L-NNA plus 10 μM INDO (n=6), L-NNA + INDO (&) + 100 μM ouabain (n=6), L-NNA + INDO (&) + 10 μM SN-6 (n=6), or L-NNA + INDO (&) + 1 μM MPP (n=6). **(B)** Summarized data shows DPN-induced CRC, R_max_, AUC and EC_50_ of male mouse mesenteric arterioles pre-constricted by NE with L-NNA plus INDO (n=6), L-NNA + INDO (&) + 100 μM ouabain (n=6), L-NNA + INDO (&) + 10 μM SN-6 (n=6), or L-NNA + INDO (&) + 3 μM PHTPP (n=6). **(C)** Summarized data shows G-1-induced CRC, R_max_, AUC and EC_50_ of male mouse mesenteric arterioles pre-constricted by NE with L-NNA plus INDO (n=6), L-NNA + INDO (&) + 100 μM ouabain (n=6), L-NNA + INDO (&) + 10 μM SN-6 (n=6), or L-NNA + INDO (&) + 1 μM G-15 (n=6). Data are expressed as a percentage of 5 μM NE induced contractions and expressed as means ± SEM. *P <0.05, **P <0.01, ***P <0.001, ****P <0.0001 and ns: no significance.


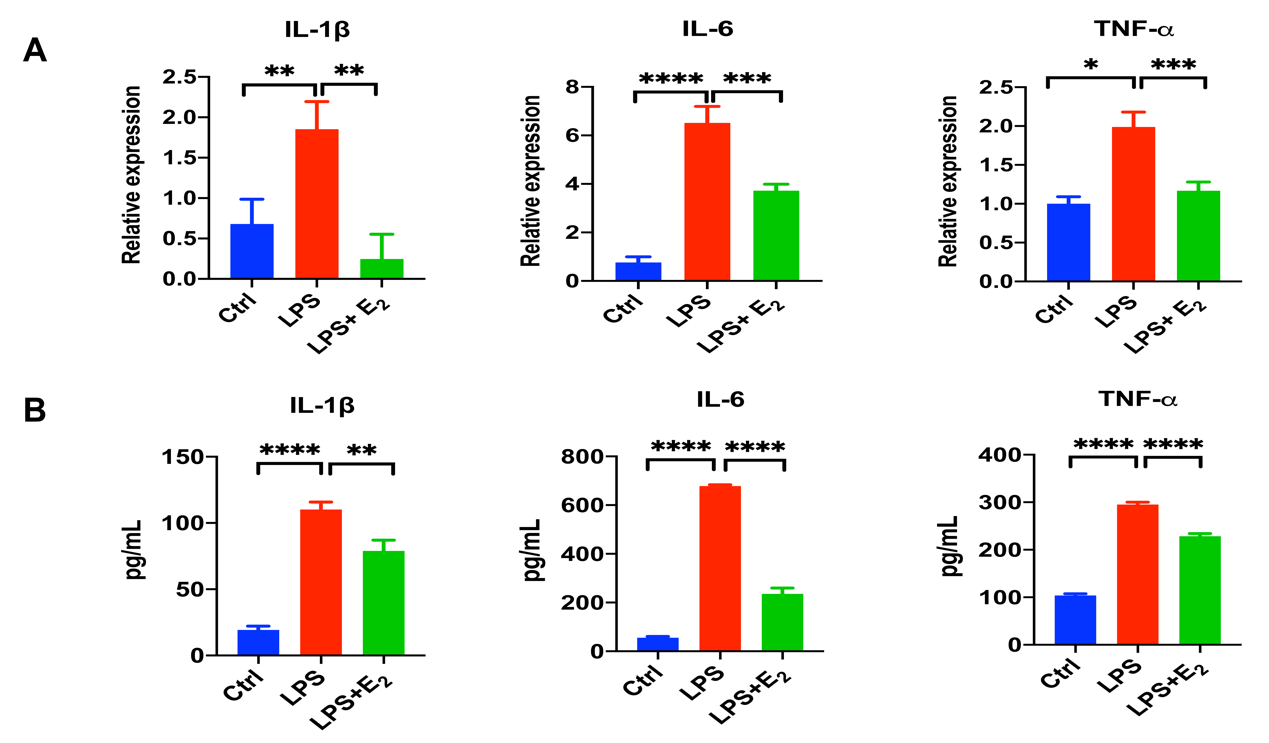


**Sup Figure 2. E_2_ reduced the expression of proinflammatory factors in LPS-induced HUVEC injury.**

**(A)** Summary data shows the mRNA expression of IL-1β, IL-6 and TNF-α of HUVEC (5 × 10^5^cells/12-well plates) after different treatments without (ctrl), or with 1 μg/ml LPS, 1 μg/ml LPS + 10 nM E_2_. **(B)** Summary data shows the protein expression of IL-1β, IL-6 and TNF-α of HUVEC (5 × 10^5^cells/12-well plates) after different treatments without (ctrl), or with 1 μg/ml LPS, 1 μg/ml LPS+ 10 nM E_2_. *P <0.05, **P <0.01, ***P <0.001, ****P <0.0001.

**
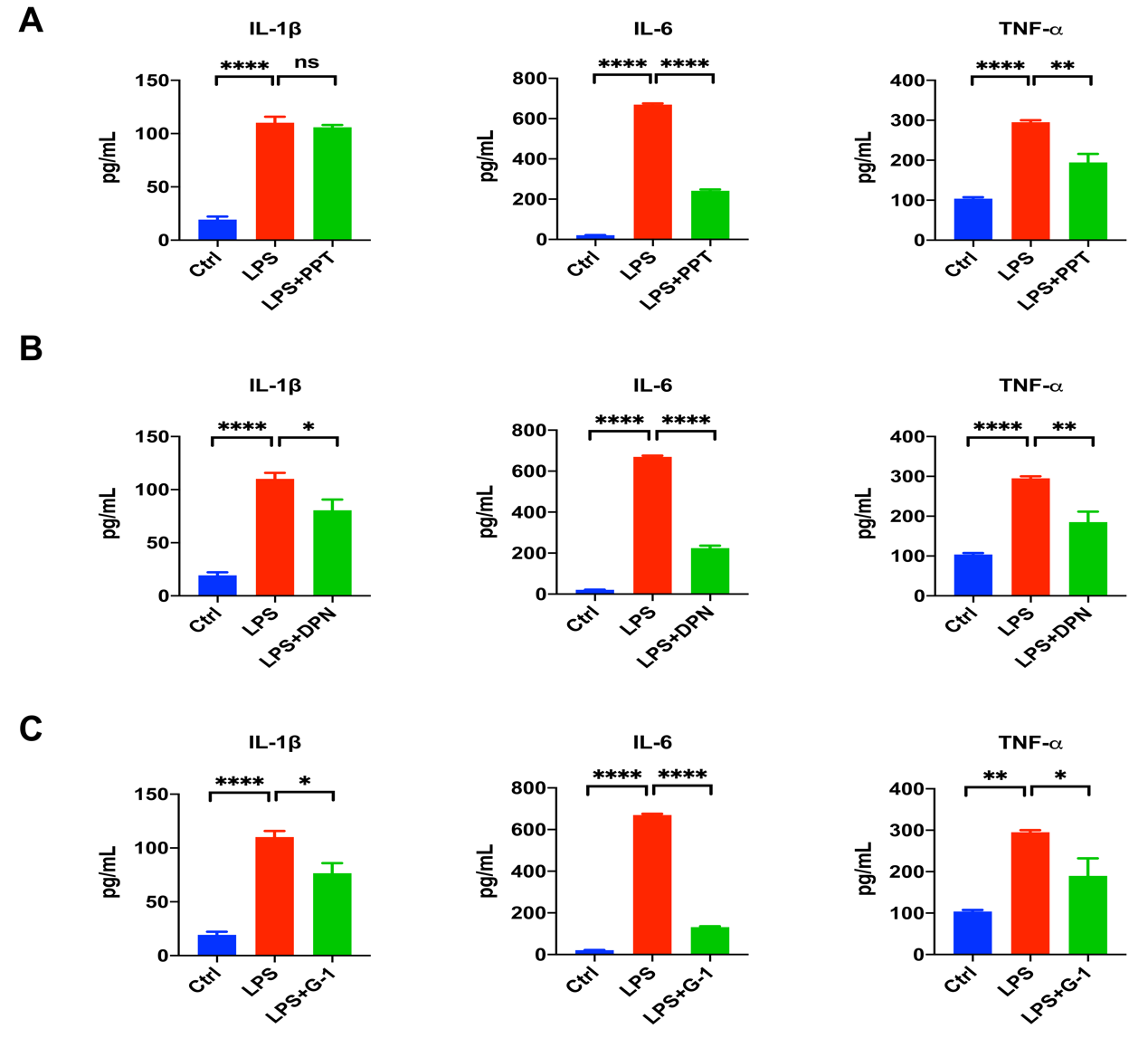
**

**Sup Figure 3. Activation of estrogen receptors reduced the expression of proinflammatory factors in LPS-induced HUVEC injury**

**(A)** Summary data shows the protein expression of IL-1β, IL-6 and TNF-α of HUVEC after different treatments without (ctrl), or with 1 μg/ml LPS, 1 μg/ml LPS + 5 nM PPT. **(B)** Summary data shows the protein expression of IL-1β, IL-6 and TNF-α of HUVEC after different treatments without (ctrl), or with 1 μg/ml LPS, 1 μg/ml LPS + 5 nM DPN. **(C)** Summary data showing the protein expression of IL-1β, IL-6 and TNF-α of HUVEC after different treatments without (ctrl), or with 1 μg/ml LPS, 1 μg/ml LPS + 1 μM G-1. *P <0.05, **P <0.01, ****P <0.0001 and ns: no significance.


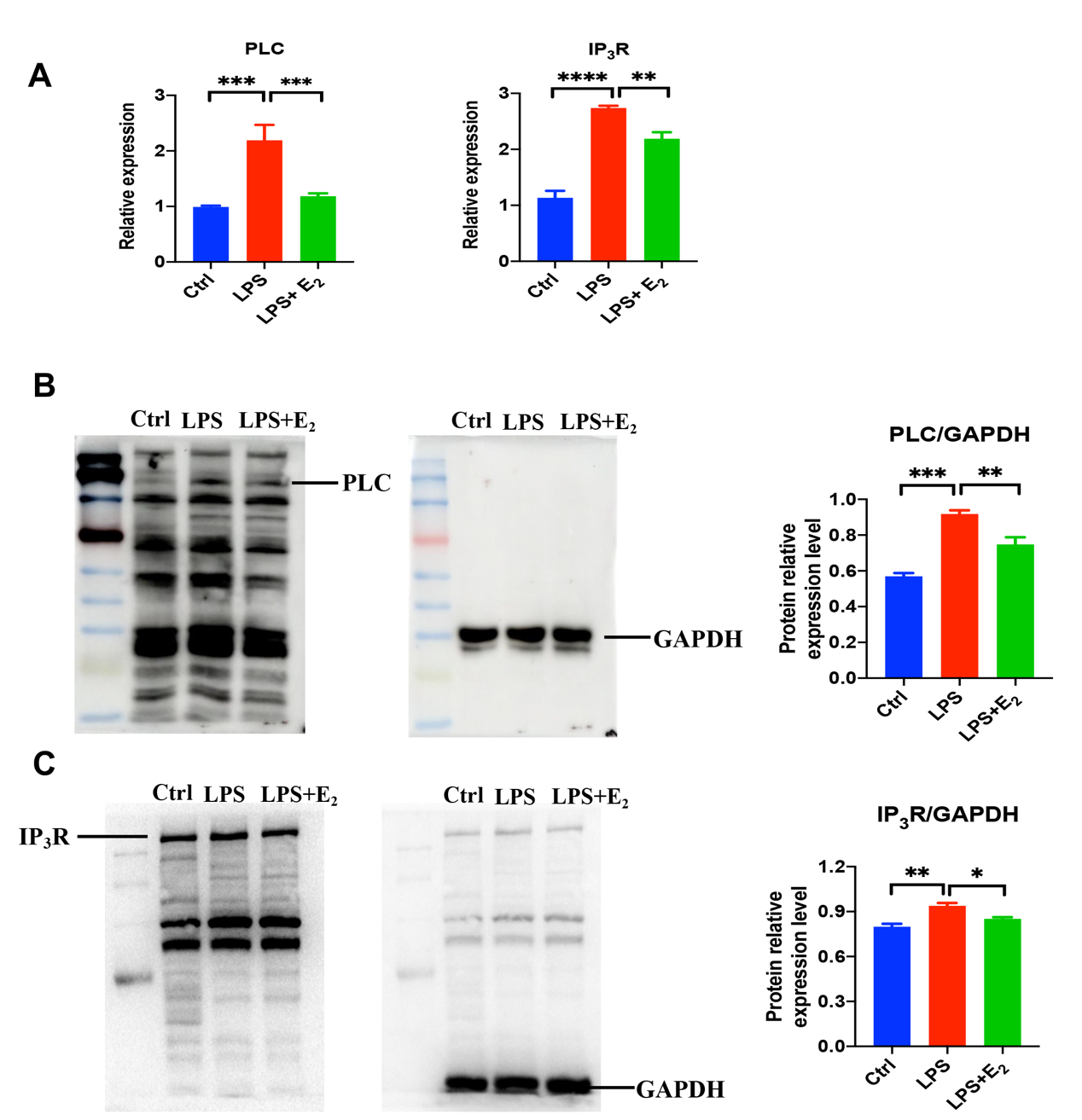


**Sup Figure 4. Confirmation of the PLC/IP_3_R pathways involved in the beneficial effects of E_2_ in LPS-induced HUVEC injury**

**(A)** Summary data shows the mRNA expressions of PLC and IP_3_R of HUVEC after different treatments without (Ctrl), or with 1 μg/ml LPS, 1 μg/ml LPS + 10 nM E_2_. (**B**) Summary data shows the protein expressions of PLC and IP_3_R of HUVEC after different treatments without (Ctrl), or with 1 μg/ml LPS, 1 μg/ml LPS + 10 nM E_2_. *P <0.05, **P <0.01, ***P <0.001, ****P <0.0001.
